# Cholesteryl ester transfer protein: ace of spades, queen of hearts, or the joker?

**DOI:** 10.3389/fphar.2015.00145

**Published:** 2015-07-14

**Authors:** Norman E. Miller

**Affiliations:** Magdalen College, Oxford UniversityOxford, UK

**Keywords:** cholesteryl ester transfer protein, CETP inhibitors, HDL, cardiovascular diseases, atherosclerosis

Plasma cholesteryl ester transfer protein (CETP) catalyzes the transfer of CEs from high-density lipoproteins (HDLs) to triglyceride-rich and low-density lipoproteins (LDLs). The hypothesis that CETP inhibition will prevent cardiovascular disease (CVD) was based on the fact that low activity increases HDL cholesterol and decreases LDL cholesterol. Early reports that CETP gene transfer increased atherosclerosis in mice, and that CETP inhibition reduced lesions in cholesterol-fed rabbits fuelled enthusiasm for the approach. Although some cautioned that the development of CETP inhibitors was premature owing to uncertainties about their impact on the remodeling of HDLs and reverse cholesterol transport (RCT) in humans (Fielding and Havel, 1996), drug discovery programmes proceeded. Two parallel research tracks then emerged. While industry progressed to clinical studies, academics sought to clarify the relations of CETP activity to RCT and atherosclerosis. Two drugs reached Phase 3 clinical trials. However, ILLUMINATE was terminated when torcetrapib was found to have had increased CVD. Five years later, Dal-OUTCOMES was aborted when it was evident dalcetrapib was not conferring any benefit.

After reviewing the literature up to May 2014, Miller (2014) concluded that CETP inhibition is more likely to increase CVD than prevent it, and was of the opinion that ongoing trials should be stopped. Since then several further pertinent studies have been published. They include four meta-analyses of the Taq1B polymorphism of the *CETP* gene. Cao et al. (2014) concluded that B2B2 homozygotes (low CETP activity, high HDL cholesterol) have a lower risk of myocardial infarction (MI) than B1B1 subjects. Using Mendelian randomization, Wu et al. (2014) found the B2 allele to be associated with a lower risk of coronary heart disease (CHD). However, another Mendelian randomization (Niu and Qi, 2015) found the B2 allele to be associated with a higher risk of CVD than the B1 allele. In this context, an earlier meta-analysis by Dullaart and Sluiter (2008) is of interest. These authors found that B2B2 carriers were less frequent among cases drawn from samples at high CVD risk than among cases drawn from population-based samples. Furthermore, in the latter case the odds ratio for CVD was 1.45 (95% CI: 1.07–1.95) in B2B2 relative to B1B1, while in the former it was 0.84 (0.74–0.96), suggesting that in the general population the B2 allele is actually associated with higher CVD risk in spite of the higher HDL cholesterol. Regieli et al. (2008) had come to a similar conclusion in the REGRESS study. After following 812 men with CHD on statins for 10 years, the B2 allele was associated with a hazard ratio for CVD death of 1.59 (*P* = 0.01) despite the expected low CETP activity and high HDL cholesterol.

Four additional meta-analyses looked at the impact of CETP inhibitors on CVD risk as part of larger studies of the effects of HDL cholesterol-raising agents in patients taking statins. All four concluded that the trials have not demonstrated a beneficial effect (Keene et al., 2014; Hourcade-Potelleret et al., 2015; Ip et al., 2015; Verdoia et al., 2015). In clinical studies, Gu et al. (2014) reported that although the A allele of the −629°C/A polymorphism of the *CETP* gene was associated with a lower plasma CETP concentration than the C allele, it had not reduced CVD events in patients taking atorvastatin. Kastelein et al. (2015) observed no significant effect of anacetrapib on CVD incidence (four events vs. zero in the treated and placebo groups, respectively) during 12 months of follow-up in patients with heterozygous familial hypercholesterolemia already on lipid-lowering treatment. Scharnagl et al. (2014) confirmed earlier reports that human plasma samples with low CETP concentrations were less effective in promoting cholesterol efflux from cultured macrophages than samples with high concentrations. In animal studies, Kühnast et al. (2015) found that anacetrapib reduced atherosclerosis in APOE^*^3Leiden.CETP transgenic mice. Briand et al. (2014) compared anacetrapib with dalcetrapib in hamsters, a species with natural CETP. In normal animals, neither drug at doses equipotent for CETP inhibition (by 60%) had any effect on macrophage-to-feces RCT, although they did lower equally the fractional clearance rate of HDL-CE. In dyslipidaemic animals, anacetrapib increased RCT, whereas an equipotent dose of dalcetrapib reduced it. Liu et al. (2015) found that inhibition of DNA topoisomerase II (Topo II) by etoposide, tenipooside or Topo II siRNA increased *CETP* gene expression and CETP secretion in HepG2 cells. When given to CETP transgenic mice, teniposide induced *CETP* expression in the liver, and increased macrophage-to-feces RCT to a greater degree than in wild-type mice with no CETP. Using a computer model of lipoprotein metabolism to analyze the on/off kinetics of the short-acting potent CETP inhibitor RG7232, Lu et al. (2015) concluded that inhibition of CETP is likely to reduce prebeta HDL production in humans.

During the past 25 years, lipidologists have become accustomed to the controversies in this field. While the decrease in LDL cholesterol has been assumed to be beneficial, the rise in HDL cholesterol has long prompted discussion on three fronts. First, the early concerns (Fielding and Havel, 1996) that HDL remodeling might be deranged, leading to lowered prebeta HDL production, have not been allayed. Second, the early assumption that the rise in HDL CE would increase the direct delivery of CEs to the liver via SR-B1 receptors was challenged by evidence that the receptors may be saturated at normal plasma HDL concentrations (Woollett and Spady, 1997; Nieland et al., 2011). Third, evidence was reported that the large CE-rich HDLs produced by CETP inhibition might be dysfunctional. Despite these concerns, momentum was maintained in the hope that the reduction of LDL would more than offset any adverse effects on HDL. However, as time has gone on the cumulative evidence has increasingly pointed to CETP having both a facilitative role in RCT and a net preventative effect on CVD. The recent work summarized above has not weakened this evidence.

Although the debate has centered largely on whether CETP inhibition is likely to have a beneficial or detrimental impact on CVD, the possibility that the net effect of the combined changes in LDL and HDL metabolism might vary according to the prevailing physiologic conditions, and therefore from subject to subject, and from time to time in the same subject, also merits consideration. Being at a crossroad in the transport of lipids, and having multiple effects on lipoprotein metabolism, the overall effects of CETP activity and its inhibition might vary according to the ambient HDL and LDL particle concentrations, for example, or to interplay with other genes. Reports of interactions between *CETP* alleles and alleles of the lipoprotein lipase (Corsetti et al., 2011), hepatic lipase (Soyal et al., 2011), apolipoprotein E (Sun et al., 2014), and nitric oxide synthase (Rahimi et al., 2012) genes seem to add weight to this possibility.

It remains that the tandem hypotheses that CETP inhibition will both enhance RCT and prevent CVD are without sound scientific bases. Indeed a growing body of evidence of three kinds now supports the contrary. First, in three out of three experimental studies of their type, macrophage-to-feces RCT *in vivo* was increased when CETP expression was enhanced (Briand et al., 2014; Miller, 2014). Second, each of five studies that examined the effect of CETP activity in human plasma on cholesterol efflux from cultured cells *in vitro* found that high activity plasma was more effective than low activity (Miller, 2014; Scharnagl et al., 2014). Third, each of six prospective cohort observational studies have found that CETP concentration or activity was related inversely to CVD incidence (Miller, 2014). In the author's opinion, this is sufficient to justify stopping all ongoing clinical trials of CETP inhibitors. Carrying on and hoping for the best, while participants are unaware of the evolving evidence base, is not acceptable.

## Conflict of interest statement

The author declares that the research was conducted in the absence of any commercial or financial relationships that could be construed as a potential conflict of interest.
